# Association of fibroblast growth factor 23 and α-klotho in hemodialysis patients during administration of ferric citrate hydrate: post hoc analysis of ASTRIO study

**DOI:** 10.1186/s12882-021-02575-9

**Published:** 2021-11-10

**Authors:** Kyoko Ito, Keitaro Yokoyama, Masaaki Nakayama, Masafumi Fukagawa, Hideki Hirakata

**Affiliations:** 1 Medical Affairs Department, Torii Pharmaceutical Co. Ltd., Tokyo, Japan; 2 Health Care Center, Harumi Toriton Clinic, The Jikei University Hospital, 1-8-8 Harumi, Chuo-ku, Tokyo, 104-0053 Japan; 3 St. Luke’s International University, St. Luke’s International Hospital, Tokyo, Japan; 4 Division of Nephrology, Endocrinology and Metabolism, Tokai University School of Medicine, Isehara, Japan; 5 Fukuoka Renal Clinic, Fukuoka, Japan

**Keywords:** ASTRIO study, Ferric citrate hydrate, FGF23, α-Klotho, Hemodialysis, iron-based phosphate binder

## Abstract

**Background:**

Fibroblast growth factor-23 (FGF23) and α-klotho are associated with anemia in patients with chronic kidney disease. In this post hoc analysis of the ASTRIO study (UMIN000019176), we investigated the relationship between FGF23 and α-klotho during treatment with an iron-based phosphate binder, ferric citrate hydrate (FC), compared with non-iron-based phosphate binders in hemodialysis (HD) patients. We examined the effect of iron absorption by FC on the relationship between FGF23 and α-klotho. There have been few clinical studies evaluating these biomarkers simultaneously in HD patients.

**Methods:**

The ASTRIO study was a 24-week, randomized, open-label, multicenter trial. HD patients taking non-iron-based phosphate binder(s) were randomized at a 1:1 ratio to continue other binder(s) (control group) or switch to FC (FC group). Serum phosphate (P) and hemoglobin (Hb) were maintained within 3.5–6.0 mg/dL and 10–12 g/dL, respectively. Plasma levels of intact FGF23 (i-FGF23), C-terminal FGF23 (c-FGF23), and α-klotho were measured, as were iron-related parameters. Association analyses of FGF23 and α-klotho were conducted.

**Results:**

Patients were randomized to FC (*n* = 48) and control (*n* = 45) groups. Serum ferritin significantly increased from baseline to end-of-treatment (EOT) in the FC group, compared with the control group (adjusted mean difference [95% confidence interval]: 79.5 [44.7, 114.4] ng/mL; *p* <  0.001). The mean change from baseline to EOT in c-FGF23 was significantly different between the FC and control groups (mean ± standard deviation (SD): − 0.2 ± 0.8 log_e_ pg/mL vs. 0.2 ± 0.8 log_e_ pg/mL, respectively; *p* = 0.04). The mean change from baseline to EOT in i-FGF23 and α-klotho were not significantly different between the FC and control groups (mean ± SD: − 0.1 ± 0.8 log_e_ pg/mL vs. 0.1 ± 0.9 log_e_ pg/mL; *p* = 0.33, and 2.0 ± 91.5 pg/mL vs. − 8.9 ± 145.3; *p* = 0.58, respectively). However, both forms of FGF23 and α-klotho were not significantly associated with each other in both groups.

**Conclusions:**

Iron absorbed via FC administration in HD patients did not influence the correlation relationship between plasma levels of FGF23 and α-klotho under the condition of serum P and Hb were maintained.

**Trial registration:**

ASTRIO study (UMIN000019176, registered at UMIN Clinical Trials Registry on October 1, 2015).

## Background

In patients with chronic kidney disease (CKD), hyperphosphatemia and anemia are common complications associated with vascular calcification and elevated mortality rates [1, 2]. Ferric citrate hydrate (FC, Riona®, Torii Pharmaceutical Co., Ltd., Tokyo, Japan) is an iron-based phosphate binder approved to treat hyperphosphatemia in patients with non-dialysis-dependent and dialysis-dependent CKD. For patients with CKD, hyperphosphatemia is the most important risk factor for vascular calcification. In the past 10 years, fibroblast growth factor-23 (FGF23) and α-klotho have been recognized as important contributors to phosphate homeostasis [3].

FGF23 is an endocrine hormone expressed in bone [4–6] that plays an important role in the maintenance of phosphate and calcium balance by binding to FGF receptors expressed in the kidney [7]. It also regulates the secretion of parathyroid hormone to ensure a normal level of serum calcium. In patients with CKD, plasma FGF23 levels increase in response to worsening kidney function. In patients with dialysis-dependent CKD, despite these higher levels of plasma FGF23, hyperphosphatemia and hyperparathyroidism are observed. Elevated plasma FGF23 is an independent risk factor for CKD progression, anemia, and reduced hemoglobin (Hb); it is also associated with cardiovascular events [8, 9]. Recently, iron homeostasis, inflammation, and erythropoiesis have been associated with FGF23 regulation [6].

Alpha-klotho (α-klotho) is a multifunctional protein expressed in the kidney, with two forms: full-length transmembrane α-klotho (mKL) and soluble secreted α-klotho (sKL) [10]. mKL acts as a coreceptor for FGF23, forming an FGF receptor-klotho complex in the kidney and parathyroid gland to increase the receptor binding affinity to FGF23 [11, 12]. This complex enhances urinary phosphate excretion while regulating phosphate and calcium homeostasis. sKL is generated by alternative splicing or cleavage from the membrane form [13, 14] and is detected in both blood and cerebrospinal fluid. sKL is presumed to have cell-protective effects including the inhibition of apoptosis, oxidative stress, and senescence [15]. The kidney is a major source of sKL; notably, reduced levels of circulating sKL are observed in older people and patients with CKD. However, it is unknown whether reduced α-klotho expression is directly caused by the absence of sKL and its protective effects; alternatively, this change in expression may be indirectly caused by the loss of mKL, which leads to elevated levels of phosphate or FGF23. The potential for α-klotho to serve as a biomarker of kidney function has been suggested in recent decades on the basis of reduced renal production of α-klotho in patients with CKD [16–18]; moreover, reduced levels of sKL are reportedly associated with the prevalence of anemia in patients with CKD [19].

In a meta-analysis in 2018, five of seven studies involving patients with non-dialysis-dependent CKD demonstrated a negative correlation between FGF23 and α-klotho [20]. Despite the known functional relationship between FGF23 and α-klotho, there have been few clinical studies evaluating these biomarkers simultaneously in patients with dialysis-dependent-CKD, as well as studies concerning the effects of drugs such as iron-containing products.

Previously, the effect of FC on renal anemia was investigated by comparing administration of FC with that of non-iron-based phosphate binders in the randomized, prospective, multicenter ASTRIO study that involved patients with CKD and hyperphosphatemia who were undergoing hemodialysis (HD) and erythropoiesis-stimulating agent (ESA) therapy [21]. In that study, the enhancement of serum ferritin from baseline to the end of treatment (EOT), reductions in ESA dose per week from baseline to EOT, and changes in plasma levels of C-terminal FGF23 (c-FGF23) from baseline to EOT were greater in the FC group than in the control group. Levels of Hb and serum phosphate (P) were maintained at 10–12 g/dL and 3.5–6.0 mg/dL, respectively, in both groups during that study. The results suggested that iron from FC was partially absorbed and contributed to the reduced dose of ESA, while improving c-FGF23 levels.

In the current study, we investigated whether iron absorbed via FC administration could influence the relationship between FGF23 and α-klotho by using data obtained from the ASTRIO study [21].

## Methods

### Study design

The ASTRIO study was a randomized, open-label, active-controlled, multicenter, parallel-arm, 24-week trial conducted at 17 medical institutions in Japan from November 2015 to January 2017 (UMIN000019176, registered at UMIN Clinical Trials Registry on October 1, 2015). Detailed methods were provided in a previous report [21]. This study was conducted in accordance with the Declaration of Helsinki. The protocol was reviewed and approved by an independent ethics committee and the Institutional Review Board of The Jikei University School of Medicine (approval number: 27–087 (7972). All participants provided written informed consent before starting the study.

### Patients

Participants were adult (age ≥ 20 years) patients with CKD who were undergoing HD for at least 12 weeks before registration, receiving one or more non-iron-based phosphate binders to treat hyperphosphatemia (monotherapy or combination therapy of sevelamer hydrochloride, lanthanum carbonate hydrate, bixalomer, and/or precipitated calcium carbonate) for at least 4 weeks before registration and were receiving an ESA (epoetin alfa, epoetin beta, darbepoetin alfa, or epoetin beta pegol) to treat renal anemia with constant dose of a single brand for at least 4 weeks before registration.

### Treatment

**Fig. 1:**
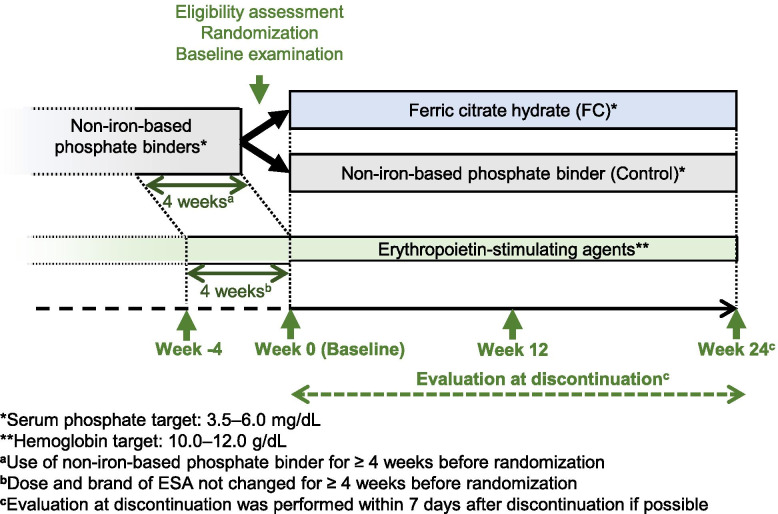
Study design. Hemodialysis patients receiving one or more non-iron-based phosphate binders and an erythropoiesis-stimulating agent (ESA) for at least 4 weeks before study registration were enrolled. Evaluation was carried out at week 0 (baseline), week 12, week 24, and at study discontinuation. End-of-treatment was at week 24 or discontinuation, whichever was earlier

Eligible patients were randomly assigned to the FC or control groups with a 1:1 allocation ratio using a permuted block method (Fig. 1).

The starting dose of FC was 1500 mg/day (two tablets of 250 mg FC, three times/day after a meal; one tablet contained approximately 60 mg ferric iron). The dose was adjusted weekly with a maximum dose of 6000 mg/day as required to maintain target serum P levels of 3.5–6.0 mg/dL in accordance with the clinical practice guidelines of the Japanese Society for Dialysis Therapy (JSDT) [22]. An ESA was administered to maintain target Hb levels of 10.0–12.0 g/dL in accordance with JSDT guidelines for renal anemia in patients with CKD [23]. All oral iron preparations other than FC were prohibited; however, intravenous iron preparations were permitted if desired by the study physician, for instance, in a case of serum ferritin < 100 ng/mL and TSAT < 20%.

### Evaluation

In the ASTRIO study, patients were evaluated for safety and efficacy at 4-week intervals until EOT (at week 24 or the day of discontinuation; Fig. 1). The primary endpoint was the mean change in ESA dose per week from baseline to EOT. The secondary endpoints included iron-related parameters at 4-week intervals and CKD-mineral bone disorder parameters, including plasma intact FGF23 (i-FGF23), c-FGF23, and α-klotho measured at baseline, weeks 12 and 24, and at study discontinuation. Blood samples were collected before hemodialysis. These values were used for post hoc analysis in the current study. Detailed safety and efficacy evaluation items were described previously [21].

The plasma levels of i-FGF23, c-FGF23, and α-klotho were determined at LSI Medience Corporation (Tokyo, Japan). Levels of i-FGF23 were measured using the FGF23 ELISA kit (Kainos, Tokyo, Japan); c-FGF23, using the FGF23 Multi-Matrix ELISA kit (Biomedica Immunoassays, Vienna, Austria); and α-klotho, using the Human Soluble α-Klotho Assay kit (IBL International GmbH, Hamburg, Germany). All other biomarkers, including serum P and Hb, were measured using a standard chemistry autoanalyzer.

### Statistical analyses

The intended sample size was estimated to be 90 patients, which provided at least 80% power to detect the difference in mean changes of ESA dose per week from baseline to EOT (the primary endpoint) between two treatment groups with a type I error of 5% according to Wilcoxon rank sum test analysis; the results of two Japanese phase III trials with FC administration for 28 and 52 weeks were used as reference data. Baseline characteristics were summarized as descriptive statistics; differences between the FC and control groups were compared using either Student’s *t*-test (continuous variables) or Fisher’s exact test (categorical variables). Mean differences in biomarker levels between the FC and control groups were evaluated using analysis of covariance with baseline values as a covariate. Associations of the plasma levels or degree of change from baseline to EOT of α-klotho vs. those of i-FGF23 or c-FGF23 in each treatment group were evaluated by calculating Pearson’s correlation coefficient. Statistical analyses were performed using SAS version 9.3 or 9.4 (SAS Institute Inc., Cary, NC, USA).

## Results

### Baseline characteristics and patient flow

**Fig. 2:**
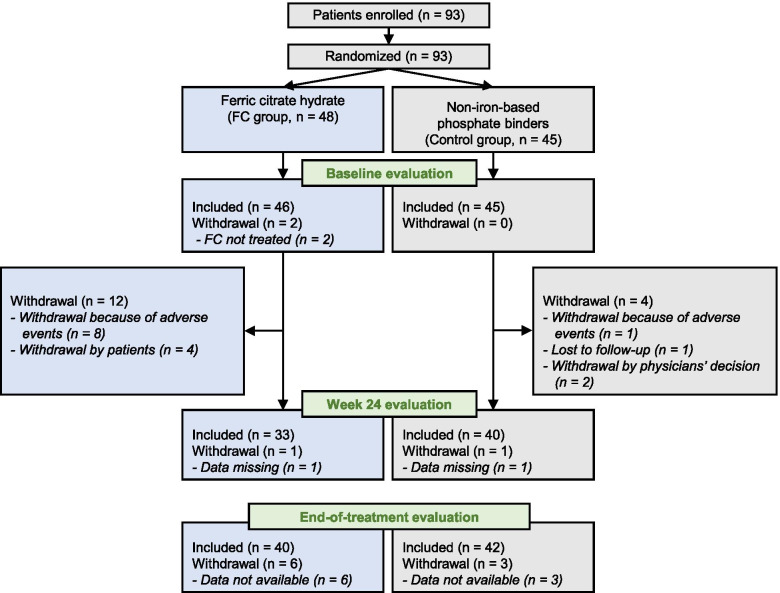
Flow of patients throughout the ASTRIO study

The recruitment period was from November 2015 to March 2016. After eligibility screening, 93 patients were enrolled and randomized (FC group, *n* = 48; control group, *n* = 45). Two patients in the FC group did not receive study treatment and 75/93 (FC group, *n* = 34/48; control group, *n* = 41/45) patients completed the 24-week study; however, one patient in each group was excluded from the analyses because of missing data. For the EOT evaluation, data from 82/93 patients were available (FC group, *n* = 40/48; control group, *n* = 42/45). The patient flow is summarized in Fig. 2.

The main baseline characteristics are summarized in Table 1. There were no significant differences in patient characteristics between the groups.

**Table 1:** Baseline characteristics of participants in the ASTRIO study (modified from Yokoyama et al., 2019 [21])

Characteristic	FC group (*n* = 46)	Control group (*n* = 45)	*p* value^*^
Age [years], mean (SD)	63.3 (10.0)	62.7 (12.7)	0.78
Body weight before dialysis [kg], mean (SD)	60.02 (10.67)	62.91 (13.61)	0.26
Male sex, n (%)	30 (65.2)	36 (80.0)	0.16
Classification of drugs for hyperphosphatemia, n (%)			
Precipitated calcium carbonate	30 (65.2)	28 (62.2)	0.83
Lanthanum carbonate hydrate	21 (45.7)	21 (46.7)	1.00
Sevelamer hydrochloride	6 (13.0)	10 (22.2)	0.28
Bixalomer	4 (8.7)	5 (11.1)	0.74
Other	0 (0)	0 (0)	–
Use of IV iron preparations, n (%)	4 (8.7)	6 (13.3)	0.52
Serum P [mg/dL], mean (SD)	5.36 (1.15)	5.15 (1.25)	0.42^**^
Hb [g/dL], mean (SD)	10.52 (0.70)	10.47 (0.94)	0.78^**^
TSAT [%], mean (SD)	23.0 (9.8)	21.2 (9.3)	0.36^**^
Serum ferritin [ng/mL], mean (SD)	105.7 (85.5)	85.6 (85.8)	0.27^**^
ESA dose^c^ [IU/week], mean (SD)	5735.4 (4933.3)	5848.1 (4082.8)	0.91^**^
i-FGF23 [pg/mL], mean (SD)	11,774.5 (14,561.0)	7883.1 (10,243.8)	0.14^**^
c-FGF23 [pg/mL], mean (SD)	1610.6 (2370.9)	1185.8 (1608.6)	0.32^**^
α-Klotho [pg/mL], mean (SD)	400.2 (107.1)	442.8 (239.1)	0.27^**^

^*^Fisher’s exact test; ^**^Student’s *t*-test; ^c^Epoetin 200 IU, darbepoetin 1 μg, and epoetin beta pegol 1 μg are equivalent

Abbreviations: *c-FGF23* C-terminal FGF23; *ESA* erythropoiesis-stimulating agent; *FC* ferric citrate hydrate; *Hb* hemoglobin*; i-FGF23* intact fibroblast growth factor-23; *IV* intravenous; *P* phosphate; *SD* standard deviation; *TSAT* transferrin saturation

### Changes in biomarkers

The changes in biomarkers from baseline to EOT are summarized in Table 2. The levels of serum P and Hb were maintained and there were no significant differences in mean level changes from baseline to EOT between the groups. Regarding iron-related parameters, mean level changes from baseline to EOT were greater in the FC group than in the control group: adjusted mean differences were 79.5 ng/mL (*p* <  0.001) in serum ferritin and 9.0% (*p* <  0.001) in transferrin saturation. The levels of i-FGF23 and c-FGF23 from baseline to EOT decreased slightly in the FC group and increased in the control group. The exponential form of the logarithmic adjusted mean difference in i-FGF23 was not significantly different between the groups (0.8; *p* = 0.33). Conversely, the exponential form of the logarithmic adjusted mean difference in c-FGF23 between the groups was statistically significant (0.7; *p* = 0.04).

**Table 2:** Changes in biomarker levels in the ASTRIO study (modified from Yokoyama et al., 2019 [21])

Variable	FC group (*n* = 40)			Control group (*n* = 42)			AMD	95% CI	*p* value^*^
	Baseline	EOT	Change	Baseline	EOT	Change			
Serum P [mg/dL]	5.36 (1.15)	5.65 (1.39)	0.24 (1.59)	5.15 (1.25)	5.04 (1.32)	−0.17 (1.53)	0.55	−0.03,1.13	0.06
Hb [g/dL]	10.52 (0.70)	10.90 (1.23)	0.45 (1.33)	10.47 (0.94)	10.74 (1.14)	0.34 (1.73)	0.17	−0.34,0.69	0.51
TSAT [%]	23.0 (9.8)	31.8 (13.6)	8.6 (12.1)	21.2 (9.3)	21.8 (10.8)	0.5 (11.8)	9.0	4.0, 13.9	< 0.001
Serum ferritin [ng/mL]	105.7 (85.5)	181.2 (108.2)	79.0 (81.5)	85.6 (85.8)	89.0 (97.4)	2.9 (79.3)	79.5	44.7, 114.4	< 0.001
i-FGF23 [log_e_ pg/mL]^**^	8.5 (1.5)	8.4 (1.5)	−0.1 (0.8)	8.1 (1.5)	8.3 (1.5)	0.1 (0.9)	0.8^***^	0.6, 1.2	0.33
c-FGF23 [log_e_ pg/mL]^**^	6.6 (1.3)	6.3 (1.5)	−0.2 (0.8)	6.3 (1.3)	6.5 (1.3)	0.2 (0.8)	0.7^***^	0.5, 1.0	0.04
α-Klotho [pg/mL]	400.2 (107.1)	399.7 (129.3)	2.0 (91.5)	442.8 (239.1)	440.3 (153.5)	−8.9 (145.3)	−11.1	−51.2, 29.0	0.58

All data are shown as mean (standard deviation)

^*^Analysis of covariance (covariate: baseline); ^**^Logarithmic transformation; ^***^Exponential form of logarithmic adjusted mean difference

Abbreviations: *AMD* adjusted mean difference (FC – Control); *Hb* hemoglobin; *c-FGF23* C-terminal FGF23; *CI* confidence interval; *EOT* end-of-treatment (day of observation at week 24 or discontinuation); *FC* ferric citrate hydrate; *i-FGF23* intact fibroblast growth factor-23; *P* phosphate; *TSAT* transferrin saturation

### Time-course changes in FGF23 and α-klotho

**Fig. 3:**
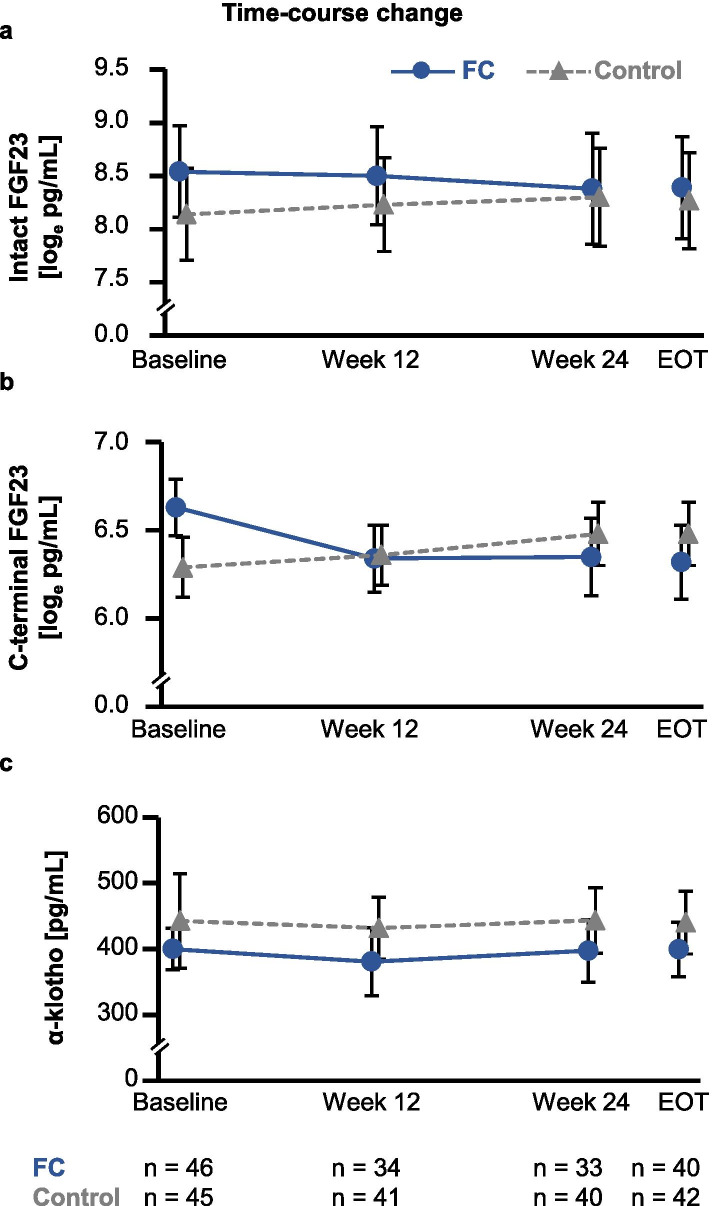
Time-course changes in levels of biomarkers. Changes in (**a**) intact fibroblast growth factor-23 (FGF23), (**b**) C-terminal FGF23, and (**c**) α-klotho. Data are presented as means and upper and lower limits of the 95% confidence interval. FC, ferric citrate hydrate; EOT, end of treatment

**Fig. 4:**
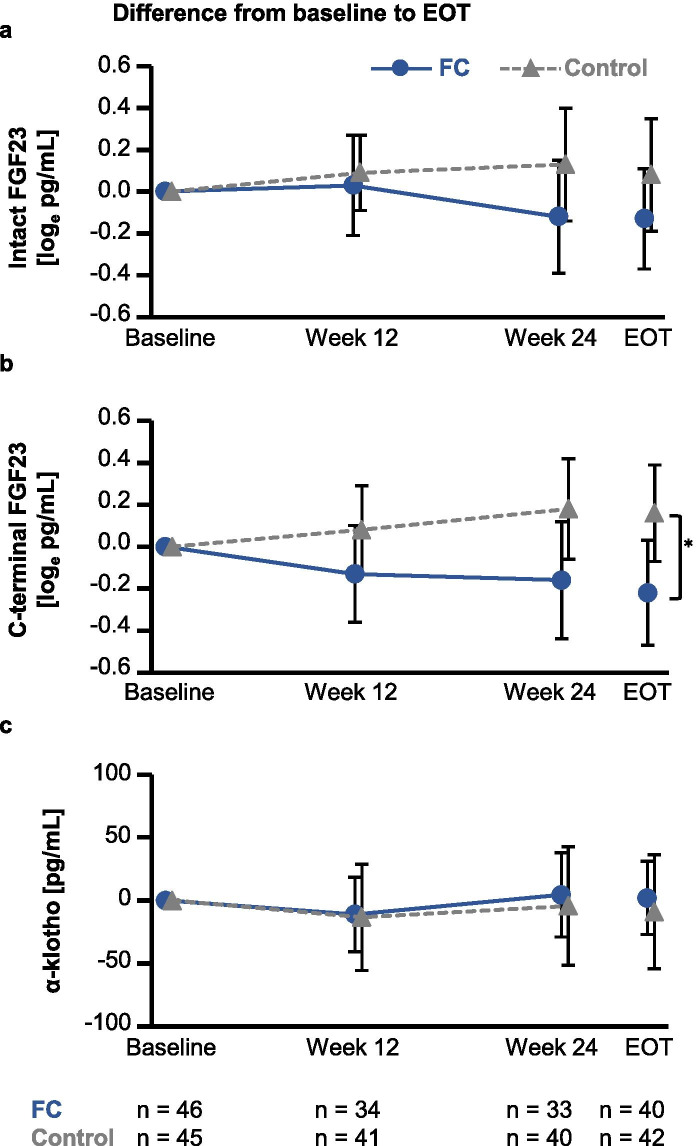
Time-course changes in difference from baseline to end-of-treatment (EOT) in levels of biomarkers. Changes in (**a**) intact fibroblast growth factor-23 (FGF23), (**b**) C-terminal FGF23, and (**c**) α-klotho. The asterisk in panel (**b**) designates a statistically significant difference between the control and FC groups (*p* = 0.04). Data are presented as means and upper and lower limits of the 95% confidence interval. FC, ferric citrate hydrate

There were no significant time-course changes in the levels of i-FGF23, c-FGF23, or α-klotho (Fig. 3). Figure 4 shows time-course changes in the difference from baseline to EOT in i-FGF23, c-FGF23, and α-klotho. The levels of i-FGF23 and c-FGF23 in the FC group tended to decrease from baseline, while those in the control group remained stable during the treatment period (Fig. 4a). The changes in c-FGF23 from baseline to EOT were significantly different between the FC and control groups (mean: − 0.2 [95% confidence interval: − 0.5, 0.0] log_e_ pg/mL vs. mean: 0.2 [95% confidence interval: − 0.1, 0.4] log_e_ pg/mL, respectively; *p* = 0.04) (Fig. 4b). There were no changes in α-klotho from baseline to EOT in either group (Fig. 4c).

### Correlation analysis

**Fig. 5:**
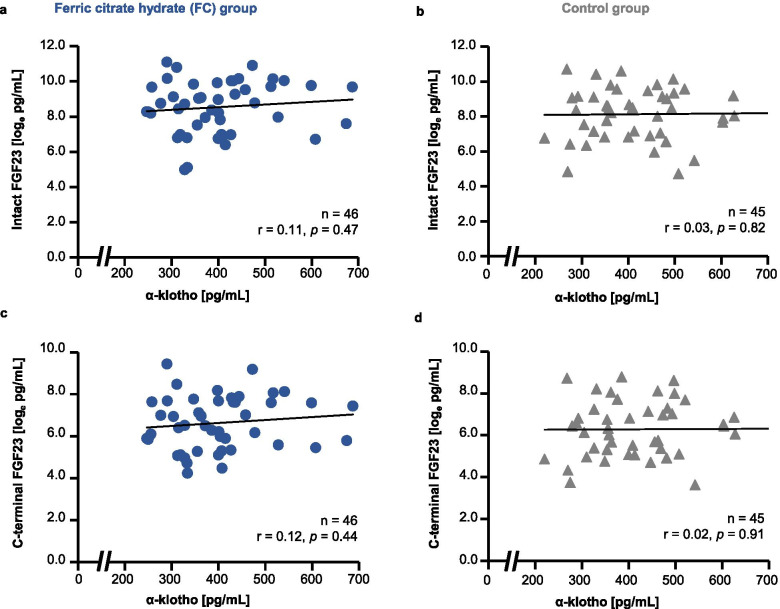
Association between baseline α-klotho levels and intact or C-terminal fibroblast growth factor 23 (FGF23). (**a**) α-klotho vs. intact-FGF23 (i-FGF23) in the ferric citrate hydrate (FC) group; **b**) α-klotho vs. i-FGF23 in the control group; (**c**) α-klotho vs. C-terminal-FGF23 (c-FGF23) in the FC group; and (**d**) α-klotho vs. c-FGF23 in the control group. FGF23 values were log-converted. r, Pearson’s correlation coefficient

**Fig. 6:**
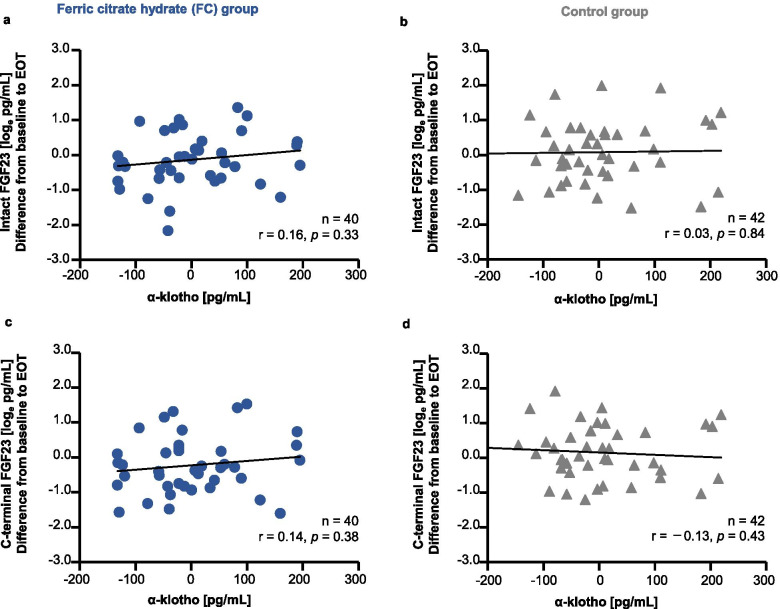
Association of change from baseline between α-klotho and intact or C-terminal fibroblast growth factor-23 (FGF23). (**a**) α-klotho vs. intact FGF23 (i-FGF23) in the ferric citrate hydrate (FC) group; (**b**) α-klotho vs. i-FGF23 in the control group; (**c**) α-klotho vs. C-terminal-FGF23 (c-FGF23) in the FC group; and (**d**) α-klotho vs. c-FGF23 in the control group. FGF23 values were log-converted. r, Pearson’s correlation coefficient

The values of α-klotho, i-FGF23, and c-FGF23 were scatter plotted to analyze associations between α-klotho and i-FGF23 or c-FGF23 in terms of baseline levels (Fig. 5) and changes from baseline to EOT (Fig. 6). At baseline, there were no significant associations: α-klotho vs. i-FGF23 in the FC group (r = 0.11, *p* = 0.47; Fig. 5a); α-klotho vs. i-FGF23 in the control group (r = 0.03, *p* = 0.82; Fig. 5b); α-klotho vs. c-FGF23 in the FC group (r = 0.12, *p* = 0.44; Fig. 5c); and α-klotho vs. c-FGF23 in the control group (r = 0.02, *p* = 0.91; Fig. 5d). Similarly, there were no significant associations in the degree of changes from baseline to EOT in any of the comparisons analyzed: α-klotho vs. i-FGF23 in the FC group (r = 0.16, *p* = 0.33; Fig. 6a); α-klotho vs. i-FGF23 in the control group (r = 0.03, *p* = 0.84; Fig. 6b); α-klotho vs. c-FGF23 in the FC group (r = 0.14, *p* = 0.38; Fig. 6c); and α-klotho vs. c-FGF23 in the control group (r = − 0.13, *p* = 0.43; Fig. 6d).

### Safety

The frequency of adverse events was similar in both groups and no serious treatment-related adverse events were observed. However, the discontinue rate due to AE were higher in the FC group (*n* = 8) than in the control group (*n* = 1) (Fig. 2).

## Discussion

FGF23 has been inversely correlated with sKL in healthy human participants [24] and patients with non-dialysis-dependent CKD [20, 25]. In addition, both FGF23 and α-klotho are reportedly associated with renal anemia [6, 19]. To the best of our knowledge, there have been few clinical studies evaluating these biomarkers simultaneously in patients with dialysis-dependent CKD. Here, we used data obtained from the ASTRIO study to evaluate the relationship between FGF23 and α-klotho during treatment with an iron-based phosphate binder (FC), compared with non-iron-based phosphate binders, in patients with CKD who were undergoing HD. In the ASTRIO study, levels of serum ferritin significantly increased and c-FGF23 significantly decreased in the FC group compared with the control group, while the serum P and Hb were maintained within the respective ranges of 3.5–6.0 mg/dL and 10–12 g/dL. Notably, we did not find any correlation relationship between FGF23 and α-klotho in either group.

The level of FGF23 reportedly increases [26], while the level of α-klotho decreases [17], with disease progression in patients with CKD. A previous randomized trial of sevelamer carbonate measured levels of FGF23 and α-klotho in patients with non-dialysis-dependent CKD; it did not show any treatment effects on the levels of i-FGF23, c-FGF23, or α-klotho in patients for whom serum P did not significantly change [27]. FC was reported to reduce FGF23 in patients with CKD who were non-dialysis-dependent [28] and those who were undergoing HD [29], when serum P significantly decreased. Among patients in the ASTRIO study who exhibited stable serum P and Hb, only c-FGF23 was significantly decreased in the FC group, compared with the control group. Taken together, these findings imply that iron absorbed by FC treatment led to reduced FGF23 production and subsequent cleavage, because c-FGF23 is reported to be increased by stimulating an FGF23 production and cleavage under the iron deficiency condition [6, 21].

The failure of FC to increase the level of sKL among patients in the ASTRIO study may be attributed to the elevated baseline levels of FGF23 in the study population. In patients undergoing HD, FGF23 levels are high [26]. In addition, initiation of HD reduced FGF23 levels but did not influence sKL levels [30], which suggests that it may be difficult to alter sKL levels in patients undergoing HD. The α-klotho protein enhances FGF23 signaling in the kidney by increasing receptor affinity [12] and regulates FGF23 synthesis in bone [31]. Therefore, klotho might be suppressed by a negative feedback mechanism induced by the high level of FGF23 in these patients. The reduction of c-FGF23 by FC in the 24-week ASTRIO study was statistically significant but may have been insufficient to stimulate klotho synthesis. A preclinical study using a rat model of ischemic acute renal failure demonstrated that ischemic lesions reduced klotho synthesis immediately, although it recovered after 4 days [32]. Whether klotho synthesis can be restored in chronically damaged kidneys in human patients is unknown and should be investigated in further studies.

Furthermore, it is tempting to speculate that FC treatment did not cause elevated α-klotho levels because the Hb level remained stable. In patients undergoing HD, the levels of sKL was reportedly positively related to Hb (*p* <  0.05) [33], and a previous 12-week study of FC compared with sevelamer hydrochloride in patients undergoing HD, the FC group significantly increased Hb compared with the sevelamer hydrochloride group (*p* < 0.001), whereas there was no difference in the change of serum P between the groups (*p* = 0.53); however, FGF23 and α-klotho levels were not evaluated in the previous study [34].

The current study had several limitations. First, the sample size of participants was small and more patients discontinued treatment due to adverse events in the FC group (eight patients) than in the control group (one patient). This difference may have been influenced by the study design, in which patients who were randomized to the FC group switched treatment from non-iron based phosphate binders to FC at baseline. Second, this study was a post hoc analysis and the sample size was calculated using a change in ESA dose per week as the primary endpoint. Therefore, the sample size may have had insufficient power to detect differences in changes of FGF23 or α-klotho, or their association. Third, the evaluation of these biomarkers was performed at three or fewer time points (baseline, weeks 12 and 24, and at study termination when applicable), which may have been inadequate for the relatively short study period. The study was also limited by its relatively short duration (24 weeks), and further evaluations may be needed. Fourth, to avoid the false discovery rate, multiple testing correction might be effective, however we have not conducted it. Finally, although the klotho protein has two forms, mKL and sKL [10], we only evaluated sKL, and the additional evaluation of mKL may have altered the results.

## Conclusions

In conclusion, when serum P and Hb were maintained at 10–12 g/dL and 3.5–6.0 mg/dL, respectively, we found that treatment with FC, an iron-based phosphate binder, let to significantly elevated serum ferritin from baseline to EOT, as well as significantly reduced ESA dose per week and plasma c-FGF23 level from baseline to EOT, compared with those parameters in patients who received treatment with non-iron-based phosphate binders. The iron absorbed during FC administration presumably contributed to anemia management and reduced FGF23 production. However, our findings showed no correlation relationship between the plasma levels of either form of FGF23 and α-klotho in patients undergoing HD.

## Data Availability

The datasets generated and/or analyzed during the study are available from the corresponding author on reasonable request.

## Abbreviations

FGF: Fibroblast growth factor
FC: Ferric citrate hydrate
EOT: End-of-treatment
c-FGF23: C-terminal fibroblast growth factor 23
i-FGF23: Intact fibroblast growth factor 23
CKD: Chronic kidney disease
Hb: hemoglobin
mKL: Transmembrane α-klotho
sKL: Secreted α-klotho
HD: Hemodialysis
ESA: Erythropoiesis-stimulating agent
P: Phosphate
JSDT: Japanese Society for Dialysis Therapy
